# Polygenic risk score for bipolar disorder associates with divergent thinking and brain structures in the prefrontal cortex

**DOI:** 10.1002/hbm.25667

**Published:** 2021-09-29

**Authors:** Hikaru Takeuchi, Ryosuke Kimura, Hiroaki Tomita, Yasuyuki Taki, Yoshie Kikuchi, Chiaki Ono, Zhiqian Yu, Izumi Matsudaira, Rui Nouchi, Ryoichi Yokoyama, Yuka Kotozaki, Seishu Nakagawa, Sugiko Hanawa, Kunio Iizuka, Atsushi Sekiguchi, Tsuyoshi Araki, Carlos Makoto Miyauchi, Shigeyuki Ikeda, Kohei Sakaki, Kelssy H. dos S. Kawata, Takayuki Nozawa, Susumu Yokota, Daniele Magistro, Tadashi Imanishi, Ryuta Kawashima

**Affiliations:** ^1^ Division of Developmental Cognitive Neuroscience, Institute of Development, Aging and Cancer Tohoku University Sendai Japan; ^2^ Department of Human Biology and Anatomy, Graduate School of Medicine University of the Ryukyus Okinawa Japan; ^3^ Department of Psychiatry, Graduate School of Medicine Tohoku University Sendai Japan; ^4^ Department of Disaster Psychiatry, International Research Institute of Disaster Science Tohoku University Sendai Japan; ^5^ Division of Medical Neuroimaging Analysis, Department of Community Medical Supports, Tohoku Medical Megabank Organization Tohoku University Sendai Japan; ^6^ Department of Radiology and Nuclear Medicine, Institute of Development, Aging and Cancer Tohoku University Sendai Japan; ^7^ Smart‐Aging Research Center Tohoku University Sendai Japan; ^8^ Department of Advanced Brain Science, Institute of Development, Aging and Cancer Tohoku University Sendai Japan; ^9^ Creative Interdisciplinary Research Division, Frontier Research Institute for Interdisciplinary Science Tohoku University Sendai Japan; ^10^ Human and Social Response Research Division, International Research Institute of Disaster Science Tohoku University Sendai Japan; ^11^ School of Medicine Kobe University Kobe Japan; ^12^ Division of Clinical research, Medical‐Industry Translational Research Center Fukushima Medical University School of Medicine Fukushima Japan; ^13^ Department of Human Brain Science, Institute of Development, Aging and Cancer Tohoku University Sendai Japan; ^14^ Division of Psychiatry Tohoku Medical and Pharmaceutical University Sendai Japan; ^15^ Department of Behavioral Medicine National Institute of Mental Health, National Center of Neurology and Psychiatry Tokyo Japan; ^16^ ADVANTAGE Risk Management Co., Ltd. Tokyo Japan; ^17^ Department of Ubiquitous Sensing, Institute of Development, Aging and Cancer Tohoku University Sendai Japan; ^18^ Center for Evolutionary Cognitive Sciences The University of Tokyo Tokyo Japan; ^19^ Research Center Institute for the Earth Inclusive Sensing Empathizing with Silent Voices, Tokyo Institute of Technology Tokyo Japan; ^20^ Faculty of Arts and Science Kyushu University Fukuoka Japan; ^21^ Department of Sport Science, School of Science and Technology Nottingham Trent University Nottingham UK; ^22^ Biomedical Informatics Laboratory, Department of Molecular Life Science Tokai University School of Medicine Isehara Kanagawa Japan

**Keywords:** bipolar disorder, creativity, polygenic risk score, prefrontal cortex, voxel‐based morphometry

## Abstract

It has been hypothesized that a higher genetic risk of bipolar disorder (BD) is associated with greater creativity. Given the clinical importance of bipolar disorder and the importance of creativity to human society and cultural development, it is essential to reveal their associations and the neural basis of the genetic risk of bipolar disorder to gain insight into its etiology. However, despite the previous demonstration of the associations of polygenic risk score (PRS) of BD and creative jobs, the associations of BD‐PRS and creativity measured by the divergent thinking (CMDT) and regional gray matter volume (rGMV) as well as regional white matter volume (rWMV) have not been investigated. Using psychological analyses and whole‐brain voxel‐by‐voxel analyses, we examined these potential associations in 1558 young, typically developing adult students. After adjusting for confounding variables and multiple comparisons, a greater BD‐PRS was associated with a greater total CMDT fluency score, and a significant relationship was found in fluency subscores. A greater BD‐PRS was also associated with lower total mood disturbance. Neuroimaging analyses revealed that the BD‐PRS was associated with greater rGMV in the right inferior frontal gyrus, which is a consistently affected area in BD, as well as a greater rWMV in the left middle frontal gyrus, which has been suggested to play a central role in the increased creativity associated with the risk of BD with creativity. These findings suggest a relationship between the genetic risk of BD and CMDT and prefrontal cortical structures among young educated individuals.

## Graphical abstract

We used brain structural imaging, genome‐wide gene polymorphism data, and creativity data as measured by divergent thinking. And we showed that a higher genetic risk of bipolar disorder is associated with higher creativity and prefrontal cortical gray matter/white matter volume in young adults.

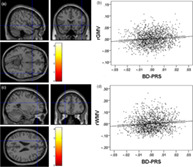

## 1 INTRODUCTION

Bipolar disorder (BD) is a mood disorder characterized by alternating states of depression and mania, resulting in psychosocial disturbances (van der Voort et al., 2015). BD shows high heritability (*h* = 0.6–0.8) (Lichtenstein et al., 2009) and BD has been reported to be highly polygenic (Craddock, Khodel, Van Eerdewegh, & Reich, 1995). A recent genome‐wide association study (GWAS) evaluating an extensive sample identified 30 loci associated with BD (Stahl et al., 2019).

It has long been suggested and accepted that BD and mania are associated with increased forms of creativity (Greenwood, 2016). Hypomania (a mild form of mania) is positively correlated with a fluency of divergent thinking (Furnham, Batey, Anand, & Manfield, 2008), which is the most typical measure of creativity in the laboratory setting. In addition, first‐degree unaffected relatives of BD patients and cyclothymic sufferers, a milder form of BD, exhibit higher creativity levels than the BD patients themselves (Richards, Kinney, Lunde, Benet, & Merzel, 1988). Given these findings, an inverted U model has been suggested (Greenwood, 2016). In this model, a moderate genetic risk of BD (moderate risk suggests the genetic risk is high, but not high enough to lead to the onset of BD) confers the advantages of positive traits, including creativity, but a high genetic risk leads to the onset of BD, which results in maladaptive psychosocial states. Indeed, BD patients exhibit greater creativity when they are in manic and mixed states than when they are in depressive states (Soeiro‐de‐Souza, Dias, Bio, Post, & Moreno, 2011). From a neuroscientific perspective, it has been hypothesized that dopamine levels are elevated to a moderately high, but not excessively high, level in manic states and individuals, and dopamine levels in the prefrontal cortex contribute to their elevated creativity (Soeiro‐de‐Souza et al., 2011).

Recent studies have focused on the neural and cognitive correlates of the overall genetic risk of BD calculated by the polygenic risk score (PRS), which is a measure of the at‐risk alleles an individual possesses. While the BD‐PRS has been shown to be associated with increased risk of major depressive disorder (MDD), the BD‐PRS is also associated with both an elevated risk of subclinical mania among individuals with MDD and a manic symptom factor among individuals with schizophrenia (Mistry, Harrison, Smith, Escott‐Price, & Zammit, 2018). In addition, using a substantial study sample, Power et al. (2015) showed that the BD‐PRS and PRS of schizophrenia were associated with artistic creativity, measured as belonging to the national artistic societies of actors, dancers, musicians, visual artists, and writers, and with higher education levels.

A wide range of neuroimaging studies investigated the neural correlates of BD‐PRS. Functional imaging studies have revealed various findings, but multiple studies have shown associations between BD‐PRS and brain activity of the right inferior frontal gyrus (IFG; Dezhina, Ranlund, Kyriakopoulos, Williams, & Dima, 2019). In addition, multiple resting state functional connectivity (RSFC) analyses have indicated that the BD‐PRS is associated with greater RSFC between the insula and other cortical areas (Dezhina et al., 2019). While structural studies with smaller sample sizes have failed to produce consistent findings in terms of structural correlates of BD‐PRS, studies based on sample sizes larger than several hundred participants have also failed to reveal any significant correlates of GMV of regions of interest within subcortical architectures and the mean microstructural property values of white matter tracts (Alemany et al., 2019; Jansen et al., 2019; Reus et al., 2017). Nonetheless, these previous studies have not considered the following issues with larger sample sizes: (a) the association between BD‐PRS and creativity measured by divergent thinking (CMDT), or (b) the association between BD‐PRS and regional GMV (rGMV)/regional white matter volume (rWMV) revealed by whole‐brain voxel‐by‐voxel analyses.

Thus, this study was undertaken to examine these issues, which are essential given the previous association study comparing BD‐PRS and creativity using creative occupation as the measured phenotype indicated the necessity of further investigation using a more refined measure of primitive creative ability (Greenwood, 2016). Divergent thinking is the most widely used measure of creative ability in the laboratory setting and predicts creative achievement (for the meta‐analysis, see Kim, 2008) and is suitable for this purpose. In addition, voxel‐by‐voxel analyses of rGMV/rWMV can identify regionally specific neural correlates of BD‐PRS in cortical structures that can help elucidate the underlying neural mechanisms of BD‐PRS (Mechelli, Price, Friston, & Ashburner, 2005).

We hypothesized that a high BD‐PRS would be associated with greater CMDT and altered rGMV/rWMV of the prefrontal cortex, given the theoretical associations between moderate genetic risk of BD, dopamine levels, and prefrontal cortex and creativity.

Given the clinical importance of bipolar disorder and the importance of creativity to the development of human society and culture, it is essential to reveal their associations and the neural basis of genetic risk of bipolar disorder to gain insight into its etiology.

## 2 METHODS

### 2.1 Subjects

The present study, a part of an ongoing project investigating the association between brain imaging and various individual differences, included 1,558 healthy, right‐handed individuals (899 males and 659 females) from whom data necessary for psychological data analyses involving polygenic scores were obtained. The mean age of the subjects was 20.77 years (*SD* 1.74; age range, 18–27 years). For detailed characteristics of the subjects, see Appendix S1. Written informed consent was obtained from all participants and **t**he study was approved by the Ethics Committee of Tohoku University.

## 3 GENOTYPING OF SUBJECTS AND CALCULATION OF PRS

Genomic DNA was extracted from saliva samples according to standard procedures. In addition, whole‐genome SNP typing was performed using the Illumina Asian Screening Array (Illumina, Inc., San Diego, California) and quality control and imputation were performed using Plink 1.9 (Chang et al., 2015) and Beagle (version 5.1) (Browning & Browning, 2009) using the 1,000 Genomes reference panel (phase 3, version 5; http://www.1000genomes.org/). For additional details of these procedures (see Appendix S1). In these processes, The principal components are calculated by Plink's command as is usually done in the field and Plink extracts top principal components from the variance‐standardized relationship matrix of the genotype data of the group and principal components are used in the following analyses.

The BD‐PRS in this study was calculated based on the clumped independent genome‐wide significant risk loci for BD which were identified in a recent genome‐wide association study of BD (Demontis et al., 2019). Thirty independent risk loci were identified using a combined analysis of discovery GWAS and follow‐up samples. The genotyped and imputed data were obtained for 24 risk loci in the present study and were used to calculate BD‐PRS (the complete list of the 24 loci, see Appendix S1). We calculated the PRS for each individual by summing the imputation probability of the reference allele of these SNPs weighted by the natural log of the odds ratio (OR) from the combined analysis of the previous study using Microsoft Excel. The equation is as follows: *w*_*i*_ is the natural log of odds ratio from the previous study for SNP_*i*_ with *w*_*i*_ = ln (OR_*i*_), and *P*_*i*_ is the probability of reference allele of each individual for that SNP. The example is presented in Figure S1.

$$\mathrm{PRS}=\sum_{i=1}^{24}w_{i}P_{i}$$

The rationale for choosing this method of PRS calculation and the results of PRS using other thresholds and PRSice‐2 (Choi & O'Reilly, 2019), are described in Appendix S1 and Figure S2.

## 4 PSYCHOLOGICAL MEASURES

Neuropsychological tests of basic cognitive performance and questionnaires for mood disorders were administered to all participants. The POMS measure and Beck Depression Inventory were used in this study to analyze the supposed basic relationship between BD‐PRS and mood states in the healthy young. The other cognitive tests were administered to see the specificity of the associations of BD‐PRS with CMDT.

Descriptions relative to this subsection were mostly reproduced from our previous studies (e.g., Takeuchi et al., 2015c) and were based on the following tests:

- Raven's advanced progressive matrix test (RAPM) (Raven, 1998), a nonverbal reasoning task which measures fluid intelligence;
- The Tanaka B‐type intelligence test (Tanaka, Okamoto, & Tanaka, 2003), a nonverbal mass intelligence test that uses figures, single numbers, and letters as stimuli, whereby the subjects have to solve as many problems as possible within a certain time (a few minutes). See Takeuchi et al. (2013) for a description of the subsets used.
- A (computerized) digit span task, which is a working memory task (for details, see Takeuchi et al., 2011).
- The S‐A creativity test (Minds, 1969; Takeuchi et al., 2010), which measures creativity through divergent thinking. Subjects are instructed to generate as many answers as possible to certain open ended questions. The SA test scores the four dimensions of the creative process (fluency, originality, elaboration, and flexibility) (Takeuchi et al., 2010). For details, see Appendix S1.
- The Stroop task (Hakoda's version) (Hakoda & Sasaki, 1990; Takeuchi et al., 2012), which measures response inhibition and impulsivity. Hakoda's version is a matching‐type Stroop task requiring subjects to check whether their chosen answers are correct, unlike the traditional oral naming Stroop task. The test consists of two control tasks, the Word–Color task and the Color–Word task, which are used as tasks for simple processing speed, a Stroop task, and a reverse‐Stroop task.
- The Japanese version (Hayashi & Takimoto, 1991) of the Beck Depression Inventory (Beck, Steer, & Carbin, 1988) was used to measure the current state of depression.
- The shortened Japanese version (Yokoyama, 2005) of the Profile Mood States (POMS) (McNair, Lorr, & Droppleman, 1992) questionnaire was used to measure the participant's mood in the preceding week. We used the total mood disturbance score, where a higher score indicates a greater total mood disturbance.

### 4.1 Image acquisition

All magnetic resonance imaging (MRI) data acquisition was performed using a 3‐T Philips Achieva scanner (Philips Healthcare, Best, The Netherlands). High‐resolution T1‐weighted structural images (T1WIs: 240 × 240 matrix, TR = 6.5 ms, TE = 3 ms, FOV = 240 mm, slices = 162, slice thickness = 1.0 mm) were collected using magnetization‐prepared rapid gradient‐echo sequences.

### 4.2 Pre‐processing of each imaging data

Preprocessing for T1‐WI for VBM analyses was performed as previously described (Takeuchi et al., 2017b) (see Appendix S1 for additional details). Briefly, the procedures were executed using the segmentation and DARTEL procedures of Statistical Parametric Mapping software 12 (SPM12; Wellcome Department of Cognitive Neurology, London, UK) to generate normalized smoothed images (8 mm full width at half maximum) of rGMV and rWMV.

### 4.3 Behavioral data analysis

Behavioral data were analyzed using R software, version 4.0.1 (R Core Team, 2014). Associations between the BD‐PRS and psychological data were obtained using multiple regression analyses. These analyses were performed considering sex, age, the six first principal components of genetic data, and the BD‐PRS as independent variables and each of the psychological measures listed in Table 2 as a dependent variable. Adjusting for several top genetic principal components is for correction for population structures in genetic association studies and a standard procedure (Price et al., 2006).

P‐values were assessed with permutation (5,000 iterations) based on multiple regression analyses using the ImPerm package (Wheeler, 2010) and R software. Linear analyses were employed as it was assumed that genetic risk of BD is associated with greater creativity until the onset of BD (Soeiro‐de‐Souza et al., 2011); this study employed a typically developing sample. For all analyses, results with a threshold of *p* < .05 (two‐sided) were considered statistically significant after correcting for the false discovery rate (FDR) using a two‐stage sharpened method (Benjamini, Krieger, & Yekutieli, 2006).

## 5 WHOLE‐BRAIN STATISTICAL ANALYSIS

Whole‐brain imaging statistical analyses were performed using SPM8 software. In the group‐level imaging analyses, we tested the associations between the BD‐PRS and regional brain volume measures (rGMV, rWMV) across the brain. For these analyses, whole‐brain multiple regression analyses were conducted, using sex, age, the first six principal components of the genetic data, and BD‐PRS as independent variables. Adjusting for several top genetic principal components is for correction for population structures in genetic association studies and a standard procedure (Price et al., 2006). We did not include total intracranial volume as a covariate. This is because the polygenic risk scores of major psychiatric disorders can have broad spread effects in whole‐brain analyses, and in such cases, regressing out global effects is improper to see the absolute regional differences of volume (Mechelli et al., 2005).

Analyses were performed including the areas of voxels with a signal intensity of >.05 for all participants. We used SPM8 instead of SPM12 due to the compatibility of the home‐made script, but the use of either SPM8 or SPM12 is not supposed to affect the results in the permutation analyses as second‐level estimation procedures of two versions return the same statistical values.

A multiple comparison correction was performed using a T score with randomized (5,000 permutations) nonparametric testing using the publicly distributed toolbox (http://dbm.neuro.uni-jena.de/tfce/). We applied a threshold of family wise error (FWE) corrected at *p* < .05.

## 6 RESULTS

### 6.1 Basic data

The mean and *SD* of age, general intelligence test scores, and BD‐PRS are presented in Table 1.

**TABLE 1:** Demographics of the study participants

Measure	Males		Females	
	Mean	*SD*	Mean	*SD*
Age	20.84	1.83	20.67	1.59
RAPM	28.79	3.88	28.00	3.89
BD‐PRS	−0.0007	0.0090	−0.0007	0.0094
S‐A creativity test—total	37.19	10.49	39.32	9.77
S‐A creativity test—fluency	34.18	9.08	35.58	8.28
S‐A creativity test—flexibility	24.81	5.28	25.80	4.82
S‐A creativity test—originality	8.36	3.31	8.14	3.15
S‐A creativity test—elaboration	28.83	8.19	31.17	7.73
BDI	7.98	6.34	8.63	6.70
POMS‐total mood disturbance	15.53	14.32	18.16	16.15

### 6.2 Psychological analyses of the correlations between BD‐PRS and individual cognitive differences

**FIGURE 1:**
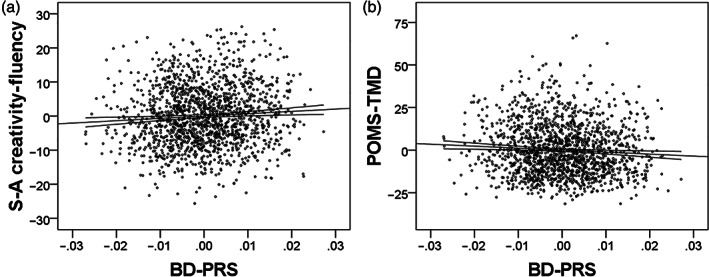
Associations between BD‐PRS and psychological variables. Partial residual plots with trend lines depicting associations between residuals of psychological variables and residuals of the BD‐PRS controlled for confounding factors. The 95% confidence intervals for the trend lines are shown. BD‐PRS was significantly associated with (a) greater fluency score of S‐A creativity test and (b) lower total mood disturbance (TMD) score of the POMS

Psychological analyses revealed that after correcting for confounding variables and multiple comparisons, the BD‐PRS significantly and positively correlated with the fluency score of the S‐A creativity test, the total score of the S‐A creativity test, and negatively correlated with the total mood disturbance score of POMS (Figure 1). The results of all statistical analyses are presented in Table 2.

**TABLE 2:** Statistical results for the multiple regression analyses investigating associations between psychological variables and BD‐PRS after correcting for confounding variables

Dependent variables	BD‐PRS				
	*N*	*β*	*t*	*p*‐value (uncorrected)	*p* (FDR)
RAPM	1,558	−0.022	−0.863	.388	.407
Total intelligence score of TBIT	1,442	−0.001	−0.046	1.000	.825
Digit span	1,548	0.008	0.324	.606	.583
S‐A creativity test—total	1,558	0.049	1.941	.010	.039
S‐A creativity test—fluency	1,558	0.071	2.822	<.0002^a^	.001
S‐A creativity test—flexibility	1,558	0.051	2.007	.050	.134
S‐A creativity test—originality	1,558	0.026	1.040	.171	.247
S‐A creativity test—elaboration	1,558	0.052	2.063	.058	.134
Word–color task	1,555	0.004	0.170	1.000	.825
Color–word task	1,556	−0.020	−0.775	.132	.218
Reverse‐Stroop task	1,554	−0.043	−1.699	.126	.218
Stroop task	1,554	−0.014	−0.549	.206	.264
BDI	1,468	−0.023	−0.870	.280	.323
POMS‐total mood disturbance	1,544	−0.070	−2.756	<.0002^a^	.001

*Note*: The table presents the *β* values, *t*‐values, uncorrected *p*‐values, and *p*‐values corrected for FDR for the multiple regression analyses that investigated associations between psychological variables and BD‐PRS after correcting for confounding variables.

^a^ For the calculation of FDR‐adjusted *p*‐values, uncorrected *p*‐values <.0002 were treated as 0.0002 (1/5,000, once in 5000 iterations).

### 6.3 Association of BD‐PRS with rGMV and rWMV

**FIGURE 2:**
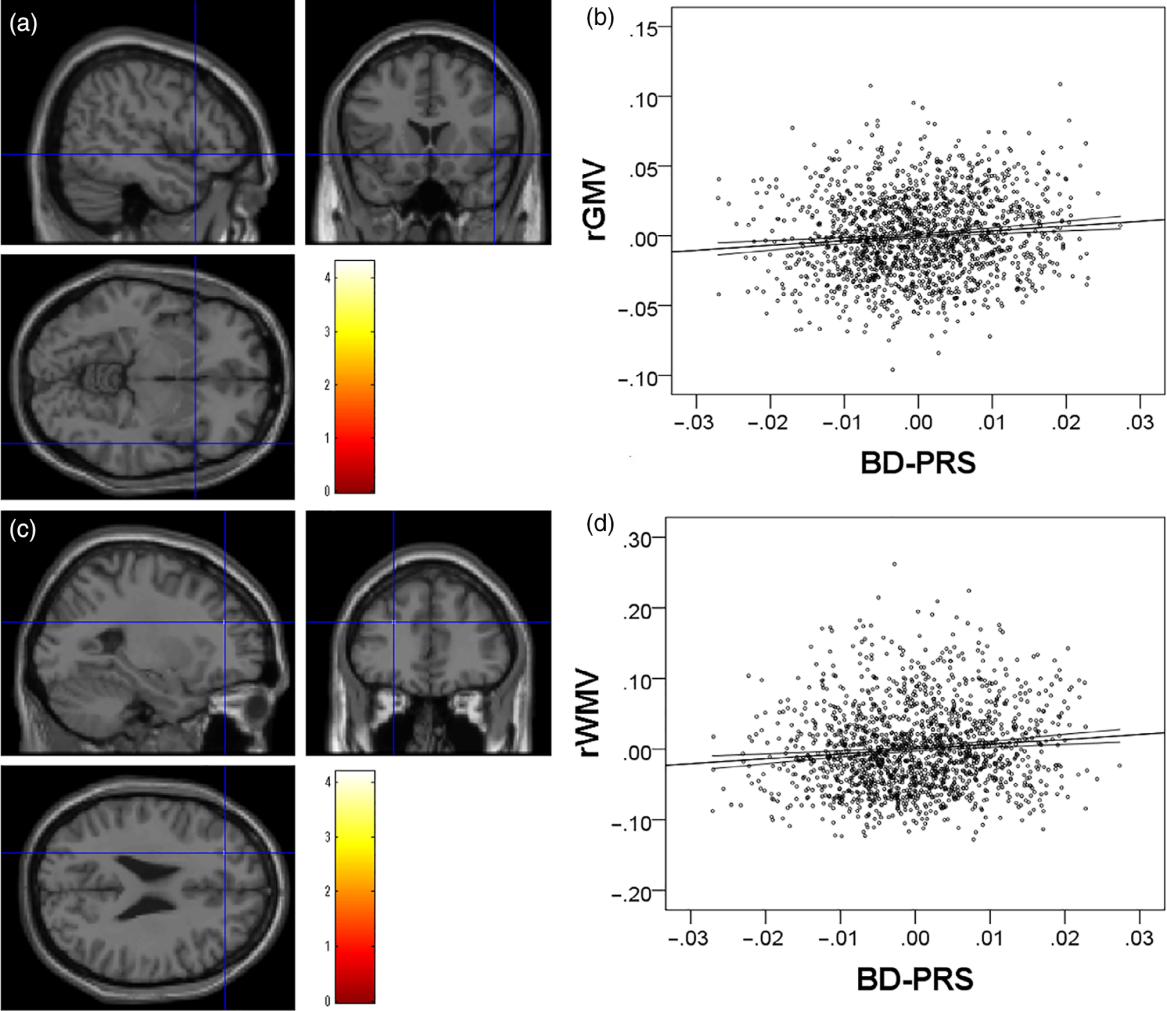
Associations between BD‐PRS and rGMV/rWMV. The BD‐PRS showed (a) a significant positive correlation with rGMV in the right IFG and (c) a significant positive correlation with rWMV in the white matter area of the left middle frontal gyrus. The correlated regions are overlaid on a SPM8 “single subject” T1 weighted structural image. In (a) and (c), the colored bars represent T scores. The results are shown using a threshold *p*‐value of <.05, corrected for multiple comparisons (permutation test using T score, F.W.E). Note, this is the stringent thresholding, so only a voxel survived the corrections for multiple comparisons, but underneath these voxels, there are many tendency level voxels (207 voxels around the significant voxel of rGMV analysis, and 63 voxels around the significant voxel of rWMV analysis with the threshold of *p* < .001, uncorrected). (b,d) Partial residual plots with trend lines illustrating associations between residuals of rGMV and rWMV values of significant clusters and residuals of BD‐PRS with controlled for other confounding factors. The 95% confidence intervals for the trend lines are shown

Whole‐brain multiple regression analysis revealed that a greater BD‐PRS was significantly associated with a greater rGMV in the right IFG area, which is in the proximity of the right anterior insula. Whole‐brain multiple regression analysis also showed that a greater BD‐PRS was significantly associated with greater rWMV in white matter areas in the middle frontal gyrus (Figure 2.) Complete statistical values are presented in Table 3.

**TABLE 3:** Brain regions with significant associations between BD‐PRS and rGMV/rWMV

Included areas	*x*	*y*	*z*	*T* value	Corrected *p*‐value (FWE)	Cluster size (voxels)
The association between high BD‐PRS and greater rGMV						
Right inferior frontal gyrus	49.5	18	−6	4.32	.050	1
The association between high BD‐PRS and greater rWMV						
Middle frontal gyrus white matter area	−25.5	39	19.5	4.19	.049	1

### 6.4 Post hoc analyses of associations between CMDT and significant neuroimaging correlates of BD‐PRS

We next investigated the associations between psychological correlates of BD‐PRS and neuroimaging correlates of BD‐PRS. Multiple regression analyses with corrections of age, sex, BD‐PRS, and six top genetic principal components revealed no significant associations between psychological correlates of BD‐PRS and neuroimaging correlations of BD‐PRS (values in the clusters of significant correlation with BD‐PRS). There were no significant correlations in any of the analyses (*p* > .1). This could be because significant correlations of CMDT are widely distributed across multiple modalities (Takeuchi et al., 2015, 2017a, 2017b, 2020). Statistical values are presented in Table 4.

**TABLE 4:** The simple correlation matrix of the associations among BD‐PRS, psychological correlates of BD‐PRS, and neuroimaging correlates of BD‐PRS

	BD‐PRS	S‐A creativity test—total	S‐A creativity test—fluency	POMS‐Total mood disturbance	Significant rGMV cluster	Significant rWMV cluster
BD‐PRS	–	0.027, .416	0.044, .192	−0.078, .020	0.120, 2.98 × 10^−4^	0.135, 4.90 × 10^−5^
S‐A creativity test—total	0.080, .04	–	0.886, 9.67 × 10^−302^	−0.015, .661	−0.058, .138	−0.037, .266
S‐A creativity test—fluency	0.112, .004	0.877, 1.56 × 10^−211^	–	−0.009, .787	−0.041, .216	−0.022, .337
POMS‐Total mood disturbance	−0.060, .126	−0.070, .075	−0.091, .020	–	−0.030, .373	−0.014, .681
Significant rGMV cluster	0.076, .051	0.014, .718	0.025, .526	0.012, .758	–	0.164, 7.88 × 10^−7^
Significant rWMV cluster	0.064, .100	0.012, .753	0.035, .376	−0.014, .720	0.186, 2.00 × 10^−6^	–

*Note*: Left values in each square are Pearson's simple correlation coefficient and right values in each square are *p* values. Upper right side squares shows the correlations in males and lower left side squares shows the correlations in females. It should be noted that neuroimaging correlates of certain variables in the whole‐brain analyses are overfitted to that variable, and such neuroimaging variables tend to correlate with other correlates of that variable (Vul, Harris, Winkielman, & Pashler, 2009).

## 7 DISCUSSION

The present study revealed an association of BD‐PRS with the psychological variable such as CMDT and neuroimaging variables, namely rGMV and rWMV with the whole‐brain voxel‐by‐voxel analyses using a large sample size. Consistent with our hypothesis, psychological analyses revealed that a higher BD‐PRS was significantly associated with CMDT, and this association was concentrated in the fluency dimension of the CMDT. Furthermore, psychological analyses also revealed that a higher BD‐PRS was significantly associated with lower total mood disturbance. In addition, neuroimaging analyses revealed that a higher BD‐PRS was significantly associated with greater rGMV in the right IFG and a greater rWMV in the left middle frontal gyrus, which was partly consistent with our hypothesis.

Our results support the idea that a moderately high genetic risk of BD is associated with high levels of creativity through greater idea fluency through good moods. We discuss the argument below. In the present study, high BD‐PRS was associated with good overall mood (i.e., less total mood disturbance) and fluency in divergent thinking. As this study was conducted using healthy subjects, a high BD‐PRS should correspond to moderately high BD‐PRS, which does not lead to the onset of BD. Previous studies have shown that certain types of good moods are associated with CMDT (Takeuchi et al., 2015b). Further, it is known that the hallmark of mania symptoms includes increased word production (Greenwood, 2016), and a milder form of mania has also been reported to be associated with the fluency of CMDT (Furnham et al., 2008). Thus, a greater BD‐PRS may lead to better mood, which may lead to greater fluency in the CMDT score.

Previous studies have reported that the BD‐PRS is associated with arts‐related creative occupations. Nonetheless, no relationships between the BD‐PRS or cognitive measures of more primitive creative potential, such as divergent thinking, have been identified, despite the proposed need for such research (Greenwood, 2016). Numerous theories have also suggested that a moderately high risk of BD is associated with higher creativity by promoting dopaminergic systems, particularly those in the prefrontal cortex. Using both brain imaging and measures of divergent thinking, the present study revealed that the BD‐PRS is associated with more complex prefrontal cortex morphology and higher CMDT fluency, which provides some answers to questions raised in previous studies.

The association between high BD‐PRS and rGMV in the right IFG region, adjacent to the right anterior insula, parallels the changes in rGMV observed in BD patients. Previous studies have also reported that the BD‐PRS was associated with brain activity during emotional tasks in the right IFG and an altered RSFC with the anterior insula (Dezhina et al., 2019). Altered activity has also been observed in patients with euthymic BD, and it has been proposed that activity depends on the affective state of BD patients (Hajek, Alda, Hajek, & Ivanoff, 2013a). The rGMV of the right IFG has been reported to be consistently larger in BD patients than in healthy controls, but this enlargement eventually decreases with the duration of the illness and has been reported to reverse following treatment with lithium (Hajek et al., 2013b). This area and the adjacent right anterior insula exert multiple functions, and the functional significance of a consistent enlargement of this area in BD patients is a matter of debate. The overuse of such functions as a form of response inhibition, which the right IFG in BD patients covers, has been suggested (Hajek, Cullis, et al., 2013b), although the robust decrease in insula volume is observed in affective disorders and may be associated with affective processing (Wise et al., 2017). Finally, this region is normally involved in word production, and the changes observed may be related to change of word production observed in BD patients (Indefrey & Levelt, 2000). However, it is difficult to conclude the causes and consequences of structural changes in this area from the current study design, and future studies are needed to investigate this issue further.

The associations of a greater BD‐PRS and greater rWMV in the left middle frontal gyrus may be comparable to the positive associations observed between the CMDT fluency and the rWMV. Herein, a higher BD‐PRS was associated with a greater rWMV in the left middle frontal gyrus area. Our previous study showed that a greater CMDT fluency was associated with greater rWMV and more widespread white matter areas, including those in the prefrontal cortex in females (Takeuchi et al., 2017b). Functional imaging studies have described the involvement of the bilateral frontal lobe in divergent thinking ability (Chávez‐Eakle, Graff‐Guerrero, García‐Reyna, Vaugier, & Cruz‐Fuentes, 2007). Creativity is a complex cognitive function, which requires many cognitive activities attributed to the prefrontal cortex (Baldo, Shimamura, Delis, Kramer, & Kaplan, 2001; Dietrich, 2004). These include working memory, attention, problem‐solving, fluency, and cognitive flexibility. Cognitive flexibility, for example, is an essential feature of developing ideas that go beyond existing frameworks and is an important element of creativity (Guilford, 1967). In addition, lesions to the frontal cortex have been reported to prohibit verbal and design fluencies (Baldo et al., 2001). The well‐developed white matter of the prefrontal cortex in high BD‐PRS subjects may support these functions and thus achieve a higher degree of creativity. The prefrontal cortex is also part of the dopaminergic system in the brain, denoted as the mesocortical system, which supports cognitive activities (Carlson, 2001). The significant results in the prefrontal cortex described in the present study may be consistent with the hypothesis that higher creativity in ones with a higher genetic risk for BD may be achieved through the moderate facilitation of the dopaminergic system, particularly the function of the prefrontal cortex (Soeiro‐de‐Souza et al., 2011). However, these inferences are speculative and need to be evaluated in experimental models that can provide more direct measures of dopaminergic activity, such as positron emission tomography.

There are a few limitations in this study to be considered. First, the study sample used in this study consisted of university students. It has been suggested that above‐average intelligence is necessary, albeit not sufficient, for higher creativity (Greenwood, 2016). Thus, although studies focusing on highly educated samples may increase the sensitivity toward detecting CMDT fluency and genetic liability toward BD, whether these findings are generalizable to other populations should be determined in future studies. In addition, we analyzed 1,558 subjects in this study with no replications of test results. Thus, although the number of participants in our study is greater than those of previous imaging studies of BD‐PRS (Alemany et al., 2019; Jansen et al., 2019; Reus et al., 2017) and the lack of replication analysis is also a standard limitation of these previous studies, future investigations are needed to replicate these findings.

Another limitation of this study is that we calculated polygenic risk scores from the previous study of European ancestry (Demontis et al., 2019). Our sample consists of a Japanese sample who can handle the Japanese language fluently. Although we are not aware of the direct commonality of the genetic basis of bipolar disorder of Japanese with that of Europeans, a recent study revealed the shared commonality of the genetic basis of European with that of another east Asian sample Han Chinese (Li et al., 2021). In addition, this previous study conducted a trans‐ancestry meta‐analysis of the genetic basis of bipolar disorder, and 16 of 30 loci that were identified in the previous study (Demontis et al., 2019) showed genome‐wide significance (though, results are driven mainly by the more prominent European sample) and two new loci. We created a polygenic risk score from these 18 loci and odds ratio of this trans‐ancestry meta‐analysis. Moreover, the correlation coefficients were similar to those of the correlation analyses of the primary analyses. For more details, see Supplemental Methods and Results.

Interestingly, a higher BD‐PRS was associated with an overall better mood in the present study sample. This result may be consistent with the fact that patients with BD showed higher creativity when they were not in the depressive state (Soeiro‐de‐Souza et al., 2011), and higher BD‐PRS being associated with higher CMDT fluency scores in the present study. Our previous study also reported that some types of better moods are associated with higher CMDT scores (Takeuchi, Tomita, et al., 2015b). Higher BD‐PRS has also been associated with a higher risk of MDD, but it was also associated with mood disorders and symptoms of mania in schizophrenia (Mistry et al., 2018). Whether BD‐PRS is associated with overall good mood as measured by POMS for this typical developmental group is not currently known. Whether subject group characteristics drive the present results, such as higher education levels, should be tested in future studies.

In conclusion, the present study describes associations between BD‐PRS with CMDT and rGMV/rWMV determined using voxel‐by‐voxel whole‐brain analyses using a large sample size. Previous studies have suggested that moderate genetic risk of BD is associated with greater creativity, and this is associated with increased facilitation of the dopaminergic system, particularly active in the prefrontal cortex. Our results provide further evidence supporting these studies in part. In our study, based on an educated typical developing sample population, greater BD‐PRS was associated with greater CMDT fluency, lower total mood disturbance, and a greater rGMV in the right IFG and rWMV in the left middle frontal gyrus.

## CONFLICT OF INTEREST

The authors declare no conflicts of interest.

## Supporting information


**Appendix S1** Supporting Information

**Figure S1** The example schema of how BD‐PRS is calculated for each individual from genotyped and imputed information of each allele and each SNP and previously shown OR for each risk allele. The equation is as follows: *w*_*i*_ is the natural log of odds ratio from the previous study for SNP_*i*_ with *w*_*i*_ = ln (OR_*i*_), and *P*_*i*_ is the probability of reference allele of each individual for that SNP.
$\mathrm{PRS}=\sum_{i=1}^{24}w_{i}P_{i}$

**Figure S2** Pearson's correlation coefficients of the correlation between two main results and the BD‐PRS of each threshold. Note among the 30 significant loci that were identified in the previous study, data from 24 loci were available in this study. The 30 significant loci in the previous study were derived from the analyses combining the discovery sample and the follow‐up sample. However, the BD‐PRS based on each *p*‐value's threshold (PT) was calculated from the data of the discovery sample of the previous study (available from the Psychiatric Genomics Consortium (https://www.med.unc.edu/pgc/).

## Data Availability

Data sharing is not applicable to this article as no new data were created or analyzed in this study.
