# Supplementary material for: Polygenic risk score for bipolar disorder associates with divergent thinking and brain structures in the prefrontal cortex
Source: Hum Brain Mapp. 2021 Sep 29;42(18):6028–37. doi: 10.1002/hbm.25667 (PMC8596941; doi:10.1002/hbm.25667)
Supplement: Supplementary file 1 — Appendix S1 Supporting Information [file HBM-42-6028-s002.docx]

**Supplemental online material**

**Supplemental Methods**

**Subjects.** The present study, which is a part of an ongoing project to investigate associations between brain imaging, cognitive function, and aging, included 1558 healthy, right-handed individuals (899 men and 659 women) from whom the data necessary for psychological analyses were successfully collected. The mean ± standard deviation [SD] age of the subjects was 20.8 ± 1.7 years (age range, 18–27 years).

Some of the subjects who took part in this study also became subjects of our intervention studies (psychological data and imaging data recorded before the intervention were used in this study)([Takeuchi, et al., 2014](#_ENREF_20)). Psychological tests and MRI scans not described in this study were performed together with those described in this study. All subjects were university students, postgraduates, or university graduates of less than one year’s standing. All subjects had normal vision and none had a history of neurological or psychiatric illness. Handedness was evaluated using the Edinburgh Handedness Inventory ([Oldfield, 1971](#_ENREF_14)). Written informed consent was obtained from each subject. For nonadult subjects, written informed consent was obtained from their parents (guardians). This study was approved by the Ethics Committee of Tohoku University.

Subjects were instructed to get sufficient sleep, maintain their conditions, eat sufficient breakfast, and to consume their normal amounts of caffeinated foods and drinks in the day of cognitive tests and MRI scans. In addition, subjects were instructed to avoid alcohol the night before the assessment.

These descriptions in this subsection were mostly reproduced from another study of ours from the same project using the exactly same methods regarding these issues ([Takeuchi, et al., 2015](#_ENREF_21)).

All subjects were either undergraduate students, graduate students or fresh graduates. All subjects had normal vision and none had neurological or psychiatric illnesses. Handedness was evaluated using the Edinburgh Handedness Inventory ([Oldfield, 1971](#_ENREF_14)).

**Details of recruitment and exclusion criteria of subjects**

They were recruited using advertisements on bulletin boards at Tohoku University or via email introducing the study. These advertisements and emails specified the unacceptable conditions in individuals with regard to participation in the study such as handedness, the existence of metal in and around the body, claustrophobia, the use of certain drugs, a history of certain psychiatric and neurological diseases, and previous participation in related experiments. They also had to be native speakers of Japanese or as fluent as native speakers (e.g., bilinguals) since the experiment was conducted using Japanese. University students, postgraduates, or university graduates of less than one year’s standing were allowed to participate, and the age was limited to 18-27 years, to control properties of participants.

A history of psychiatric and neurological diseases and/or recent drug use was assessed using our laboratory’s routine questionnaire, in which each subject answered questions related to their current or previous experiences of any of the listed diseases and listed drugs that they had recently taken. Drug screening was performed to confirm that the subjects were not taking any illegal psychostimulants or antipsychotic drugs, which was one of the exclusion criteria used during the course of the recruitment. Subjects with exclusion criteria should have been excluded before they came to the lab, but if they came for some reason, they had to go back once it was found that they met an exclusion criterion. Consequently, none had a history of neurological or psychiatric illness. In the course of this experiment, the scans were checked for obvious brain lesions and tumors, but there were no subjects having such obvious lesions or tumors.

These descriptions are mostly obtained from our previously published work ([Takeuchi, et al., 2021](#_ENREF_26)).

**Details of genotyping.** Saliva samples from participants were collected using Oragene containers (DNA Genotek Inc., Ottawa, Canada), and high-molecular-weight DNA was extracted using the Oragene saliva DNA extraction kit (DNA Genotek Inc., Ottawa, Canada) according to the manufacturer’s protocol at Tohoku University. DNA samples were then forwarded to Riken Genesis Co., Ltd. (Yokohama, Kanagawa, Japan) and Tokai University for whole-genome SNP typing using the Illumina Asian Screening Array (Illumina, Inc., San Diego, CA, USA).

DNA samples were denatured and neutralized under alkali conditions. The denatured samples were then amplified by whole-genome amplification (37°C overnight). Amplified DNA samples were enzymatically fragmented for 1 hour at 37°C in a microsample incubator and 2-propanol was added to the fragmented DNA samples and precipitated by centrifugation. Precipitated DNA samples were re-suspended with hybridization buffer and incubated for 1 hour at 48°C in a hybridization oven. Fragmented and re-suspended DNA samples were denatured for 20 minutes at 95°C in a microsample incubator. Denatured DNA samples were dispensed onto BeadChips using the TECAN System. The BeadChips were incubated overnight at 48°C in an hybridization oven to hybridize the samples onto the BeadChips. After hybridization, seals were removed from the Hybridized BeadChips and unhybridized fragment DNAs were washed away. Labeled nucleotides were added to the washed BeadChips to extend the primers that had hybridized to the DNA. BeadChips were stained, coated for protection, and then dried. The dried BeadChips were scanned by iSCAN System.

Data analysis was performed as follows. Signal intensity data (idat files) generated in previous step using Illumina GenomeStudio 2.0 software were analyzed by applying the Illumina cluster file (ASA-24v1-0_A1_ClusterFile.egt) and initial SNP genotypes were called. The genotypes were refined using the GenomeStudio clustering function. (GenTrain3.0 clustering algorithm was used).

**Details of quality control of the genotyped data and imputation**

Quality control of the genotype was conducted using PLINK (v1.9) ([Chang, et al., 2015](#_ENREF_3)). We excluded markers with high missingness rates (>10%), Hardy-Weinberg disequilibrium (HWE, P < 1 × 10^−6^) and extracted only markers in autosomes. We excluded individuals with heterozygosity greater than 0.065 based on SNPs with minor allele frequency >0.01, genetic and phenotypical sex mismatches, relatedness with other individuals (one of each pair with a genetic relationship greater than 9.375% [the midpoint between 1/8 and 1/16] was excluded) and genotype quality (<10% missing), and 5 SD than values of the 3 largest principal components. We performed imputation using the 1000 genomes dataset (phase 3, version 5; http://www.1000genomes.org/) as a reference panel using Beagle (version 5.1) ([Browning and Browning, 2009](#_ENREF_2)). Before the imputation, SNP ID duplicates and SNPs with irregular alleles of insertion (I) and deletion (D) from were removed from our own data and the strand correction was performed using the conform-gt program with East Asian subsets of the 1000 genomes (JPT and CHB). SNPs with DR2 values (dosage R-squared) below 0.8 and SNPs with minor allele frequency <0.01 were removed from the imputed data.

Based on the recommendation of the expert of the genetic studies (Ryosuke Kimura, Second author), we chose to use the East Asian subsets of the 1000 genomes (JPT and CHB, (phase 3, version 5)) for the strand check. We chose these subsets because in this process, we use frequency information. However, we chose to use all subsets of the 1000 genomes for the imputation procedure. When SNPs with minor allele frequency are excluded, imputation using the larger sample size, including distant ancestries, is shown to have a better performance ([Hancock, et al., 2012](#_ENREF_6)). Because haplotypes are shared between populations, individuals with a different ancestry background are informative for the imputation.

In this study, we removed outliers in 3 top genetic principal components from analyses and added 6 top genetic principal components in the multiple regression analyses. Removal of outliers in the first few principal components is common practice ([Kässens, et al., 2021](#_ENREF_7)), and adding more principal components is also a standard procedure ([Reed, et al., 2015](#_ENREF_17)). We followed these procedures. However, the selection of how many principal components is chosen varies between studies and seems somewhat arbitrary. However, even if we add just 3 top principal components in the multiple regression analyses, the results are affected little.

**The 24 genome-wide significant loci used in this study**

Among the 30 genome-wide significant risk loci of bipolar disorders (BD) described in a previous study ([Stahl, et al., 2019](#_ENREF_19)), individual genotyped and imputed data for the following 24 risk loci were available after imputation and were used in the calculation of PRS in the main analysis.

rs57195239, rs17183814, rs61332983, rs9834970, rs2302417, rs3804640, rs11724116, rs10035291, rs57970360, rs2388334, rs10455979, rs113779084, rs73188321, rs10994318, rs59134449, rs10896090, rs7122539, rs12575685, rs10744560, rs139221256, rs11647445, rs112114764, rs11557713, rs111444407.

**Rationale for choosing the abovementioned BD-PRS calculation method and calculation of BD-PRS using more lenient thresholds**

The 30 genome-wide significant SNPs defined in a previous study ([Stahl, et al., 2019](#_ENREF_19)) were identified in the analysis using the combined discovery sample and follow-up sample. The BD-PRS in the main analysis was calculated based on these SNPs.

This was primarily because we first aimed to take advantage of these newly identified genome-wide significant loci of BD risk ([Demontis, et al., 2019](#_ENREF_5)). Additionally, the following supplemental analysis showed BD-PRS calculated from this method showed the highest association with the criterion measure (Total mood disturbance score of POMS). Finally, using this method, we could see if the tendency of the results changed if we used the loci identified from the trans-ancestry meta-analysis *(*[*Li, et al., 2021*](#_ENREF_9)*),* including the East Asian sample as described in the following subsection. Although it is a relatively new method to use genome-wide significant loci to calculate PRS, as identification of lots of genome-wide significant loci has only begun recently, it is also taken in several studies. While it is known which threshold is best for the calculation of PRS seem highly variable depending on the measures, many studies showed the genome-wide significant loci explain the variance best ([Miller, et al., 2018](#_ENREF_10); [Prins, et al., 2016](#_ENREF_15); [Ranlund, et al., 2018](#_ENREF_16)).

However, we also calculated BD-PRS based on more lenient thresholds. Note that in these analyses, the summary statistic data of the discovery sample from the previous study ([Stahl, et al., 2019](#_ENREF_19)) were used (available from the Psychiatric Genomics Consortium (<https://www.med.unc.edu/pgc/>).

The BD-PRS based on the lenient thresholds were generated using PRSice-2 (<https://www.prsice.info/>). From the summary statistics data, we removed SNPs with low info-score <0.8, SNPs with MAF <0.01, and ambiguous SNPs. The criterion for SNP clumping was a pairwise linkage disequilibrium of R^2^ less than 0.25 within 200 kb windows. We calculated the PRS scores with different P-value thresholds, ranging from 5 × 10^−8^ to greater than 1.0. The dosage data was used for these calculations of PRS. The hard-threshold was set to 0.

**Supplemental analyses using the BD-PRS derived from the 18 significant loci in the previous trans-ancestry analysis**

In addition, we calculated another polygenic score from the significant loci and odds ratio of previous trans-ancestry meta-analysis of the risk of BD from the sample of [Demontis, et al. (2019)](#_ENREF_5), and East Asian (Han Chinese)*(*[*Li, et al., 2021*](#_ENREF_9)*)*. This meta-analysis yielded 18 significant loci (16 are common to the study of [Demontis, et al. (2019)](#_ENREF_5), and the result seems mainly driven by the sample of [Demontis, et al. (2019)](#_ENREF_5)). And then, we compared the correlation coefficients of these associations with significant correlations of BD-PRS calculated from 24 significant loci, presented in the main text. The details and 18 loci were described in the previous study*(*[*Li, et al., 2021*](#_ENREF_9)*)*.

**Details of the S-A creativity test**

The descriptions in the S-A creativity tests are largely reproduced from our previous study using the same methods ([Takeuchi, et al., 2020](#_ENREF_22)).

The S-A creativity test ([Minds, 1969](#_ENREF_11)) was used for assessing creativity measured by divergent thinking.

This test involves three types of tasks: Practice (and real) tasks were administered in the following order: (1) practice of the first task (2 min), (2) first task (5 min), (3) practice of the second task (2 min), (4) second task (5 min), (5) practice of the third task (2 min), and (6) third task (5 min). Each task involves two questions. In total, the test takes 30 min. How subjects divided their time (5 min in total) for two questions was not determined. The 2 questions were presented on 2 facing pages, and on each page there were also 10 lines under the question on which subjects were required to write down self-generated answers.

This test was administered in a group setting. The first task requires subjects to generate unique ways of using typical objects (e.g., “Other than for drinking milk, how can we use milk bottles?” Example answer: “We can use them as saving boxes.”). The second task requires subjects to imagine desirable functions of ordinary objects (e.g., “What are the characteristics of a good TV? Write down as many characteristics as possible.” Example answer: “A TV can receive broadcasts from all over the world.”). The third task requires subjects to imagine the consequences of “unimaginable things” happening (e.g., “What would happen if all the mice in the world disappeared?” Example answer: “The world would become more hygienic.”). For each task, subjects are required to generate as many answers as possible. Note that these tasks correspond with the three tasks (unusual use, product improvement, just suppose) of the Torrance test of creative thinking ([TTCT; Torrance, 1966](#_ENREF_27)), which is used in other countries.
Scoring was performed by the Tokyo Shinri Corporation. In addition to a total score, the S-A creativity test provides subscores for the following dimensions of creativity: (a) Fluency: Fluency is measured by the number of relevant responses to questions and is related to the ability to produce and consider several alternatives. Fluency scores are determined by the total number of questions answered after excluding inappropriate responses or responses that are difficult to understand. (b) Flexibility: Flexibility is the ability to produce responses from a wide perspective. Flexibility scores are determined by the sum of the (total) number of category types to which the responses are assigned based on a criteria table or similar judgment. (c) Originality: Originality is the ability to produce ideas that differ from those of others. For originality scoring, each answer was assigned to an idea category from a criteria table or similar judgment. Each category received different originality points based on appearance frequencies, and originality score was calculated as the sum of all these points. In the case of the first task, answers categorized to “containers” had high appearance frequencies (>5%) and so were awarded 0 points. Alternatively, the answers categorized as “alternatives for musical instruments” had lower appearance frequencies (1%−5%) and so were awarded 1 point, while rarer answer categories or answers that could not be categorized were awarded 2 points. (d) Elaboration: Elaboration is the ability to produce detailed ideas (Society for Creative Mind, 1969). Elaboration scores are determined by the sum of responses weighted based on a criteria table or similar judgment. In the case of the first task, answers that were classified as the lowest level of elaborateness, “unclear answers” such as “musical instruments” (within the “alternatives for musical instruments” category), were awarded 0 points, while answers classified to the middle level of elaborateness, which have typically only means or purposes such as “beat and make sounds” were awarded 1 point, and answers classified as the highest level of elaborateness, which have typically both means and purposes and/or more details such as “arrange milk bottles in a row and put different amounts of water in each bottle and beat to use as instruments” were awarded 2 points. Again, these four dimensions correspond to the TTCT ([TTCT; Torrance, 1966](#_ENREF_27)). Scoring of the tests was performed by the Tokyo Shinri Corporation.

In the present study, total score and each dimension score were used. The total score is the sum of the originality score and elaboration in the S-A creativity test ([Minds, 1969](#_ENREF_11)) used here (as stipulated by the manual of this test) and is also called as overall score. The average z scores of the four dimensions and this total score (originality + elaboration) were highly correlated (r = 0.97).

Please refer to the appendix of our previous study for a sample test and additional details on scoring ([Takeuchi, et al., 2010](#_ENREF_24)).

Each subfactor of the S-A creativity test scores was significantly correlated with other external measures, such as personality factors and problem-solving abilities, suggesting its ability to predict performance in everyday situations ([Shimonaka and Nakazato, 2007](#_ENREF_18)). Furthermore, S-A creativity test scores (total score) are significantly correlated with the frequency of visual hypnagogic experiences, which in turn is correlated with the vividness of mental imagery ([Watanabe, 1998](#_ENREF_28)). Our previous study ([Takeuchi, et al., 2013](#_ENREF_25)) showed that S-A creativity test scores (total score) were positively correlated with extraversion, novelty seeking, motivational state, and daily physical activity level, which are consistent with reports for other measures of CMDT ([Chavez-Eakle, et al., 2006](#_ENREF_4); [King, et al., 1996](#_ENREF_8)). The total score on the S-A creativity test was positively correlated with trait creative attitude as measured by self-report in children ([Nish and Niwase, 2003](#_ENREF_12)), with scores on a modified version of the figure completion test of figural TTCT in children ([Ogata, 1976](#_ENREF_13)), and with performance on a novel problem-solving task ([Ogata, 1976](#_ENREF_13)). Each subfactor of the two S-A creativity test tasks was positively correlated with each subfactor of each originally developed chemical divergent thinking creativity test task (e.g., How can you prevent ice which is taken from the refrigerator from melting?) ([Wulanqiqige, 2014](#_ENREF_29)).

**Details of preprocessing methods used for T1-weighted structural images for VBM analyses**

Preprocessing of the structural data was performed using Statistical Parametric Mapping software (SPM12; Wellcome Department of Cognitive Neurology, London, UK) implemented in Matlab (Mathworks Inc., Natick, MA, USA). Using the new segmentation algorithm implemented in SPM12, T1-weighted structural images of each individual were segmented into 6 tissues. In this new segmentation process, default parameters were used, except that the Thorough Clean option was used to eliminate any odd voxel, affine regularization was performed with the International Consortium for Brain Mapping template for East Asian brains, and the sampling distance was set at 1 mm. We then proceeded to the diffeomorphic anatomical registration through exponentiated lie algebra (DARTEL) registration process implemented in SPM12. We used DARTEL import images of the 2 TPMs from the abovementioned new segmentation process. First, the template for the DARTEL procedures was created using imaging data from 800 participants (400 males and 400 females). Next, using this existing template, the DARTEL procedures were performed for all of the subjects in the present study. In these procedures, default parameter settings were used. The resulting images were spatially normalized to the Montreal Neurological Institute (MNI) space to generate rGMV and rWMV images with 1.5 × 1.5 × 1.5 mm^3^ voxels. In addition, we performed a volume change correction (modulation) by modulating each voxel with the Jacobian determinants derived from spatial normalization, which allowed us to determine regional differences in the absolute amount of brain tissue ([Ashburner and Friston, 2000](#_ENREF_1)). Subsequently, all images were smoothed by convolving them with an isotropic Gaussian kernel of 8 mm full width at half maximum (FWHM).

The descriptions in this subsection were mostly reproduced from our previous study using the same methods ([Takeuchi, et al., 2017](#_ENREF_23)).

**Supplemental Results**

**Supplemental analyses using the BD-PRS derived from the 18 significant loci in the previous trans-ancestry analysis**

The changes of statistical values of BD-PRS from that of BD-PRS calculated from the European sample (those in the main text) to that of BD-PRS calculated from the trans-ancestry meta-analysis are as follows POMS-TMD (β = −0.070, p (permutation) = <0.0002 -> β = −0.075, p (permutation) = <0.0002), SA-creativity test total score (β = 0.049, p (permutation) = 0.010 -> β = 0.055, p (permutation) = 0.0024), SA-creativity test fluency (β = 0.071, p (permutation) = <0.0002 -> β = 0.063, p (permutation) = 0.0008), and rGMV of significant area of the whole gray matter analysis (β = 0.104, p = 0.00002 -> β = 0.082, p = 0.001) and rWMV of significant area of the whole rWMV analysis (β = 0.100, p = 0.00003 -> β = 0.084, p = 0.0005).

**Supplemental Figure legend.**

**Supplemental Fig. 1.** The example schema of how BD-PRS is calculated for each individual from genotyped and imputed information of each allele and each SNP and previously shown OR for each risk allele. The equation is as follows: *w_i_* is the natural log of odds ratio from the previous study for SNP*_i_* with *w_i_* = ln (OR*_i_*), and *P_i_* is the probability of reference allele of each individual for that SNP.

24

PRS = ∑w_i_P_i_

_i_ = 1

**Supplemental Fig 2.** Pearson’s correlation coefficients of the correlation between two main results and the BD-PRS of each threshold. Note among the 30 significant loci that were identified in the previous study, data from 24 loci were available in this study. The 30 significant loci in the previous study were derived from the analyses combining the discovery sample and the follow-up sample. However, the BD-PRS based on each P-value’s threshold (PT) was calculated from the data of the discovery sample of the previous study (available from the Psychiatric Genomics Consortium (<https://www.med.unc.edu/pgc/>).

**Supplemental Fig 1.**


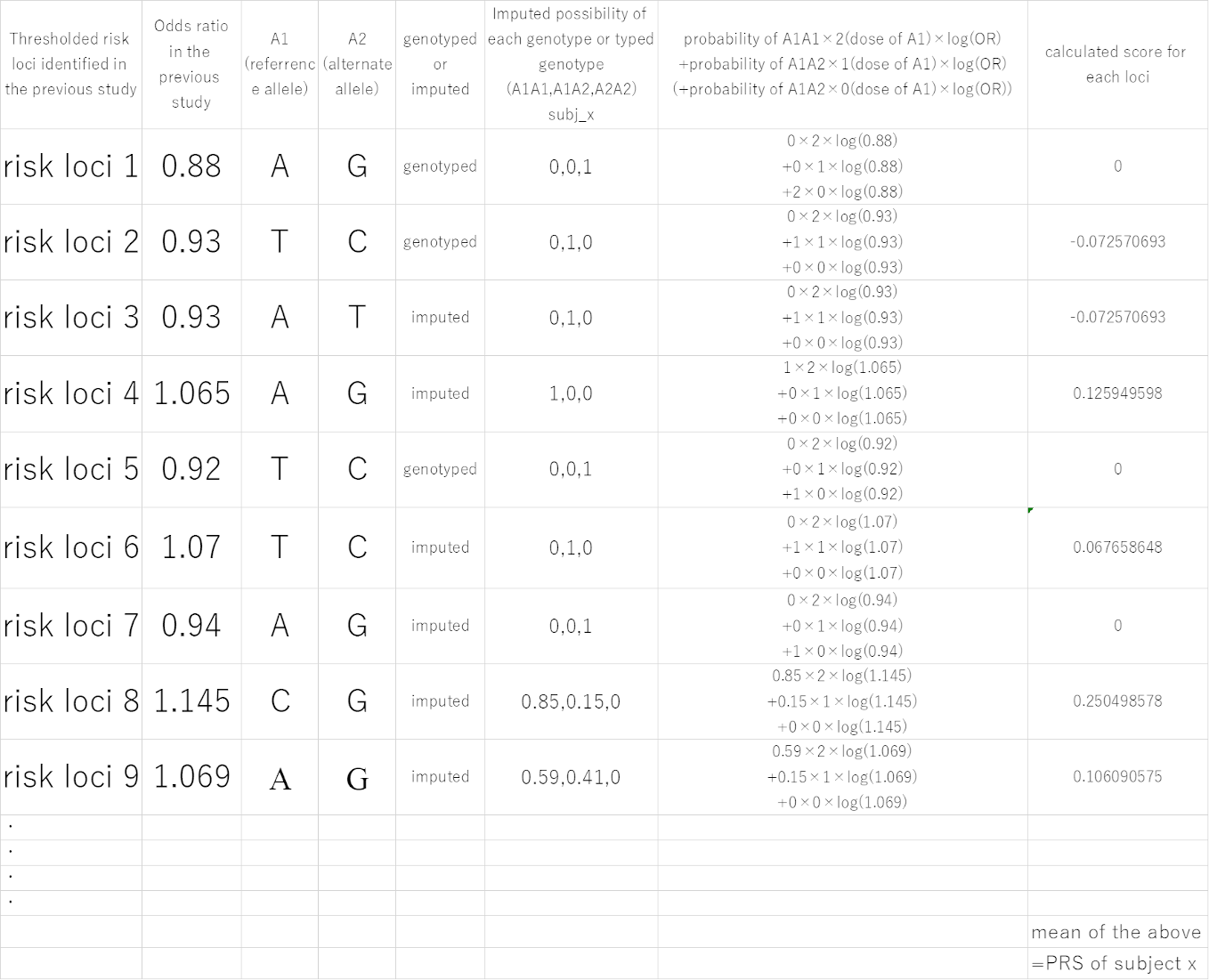


**Supplemental Fig 2.**

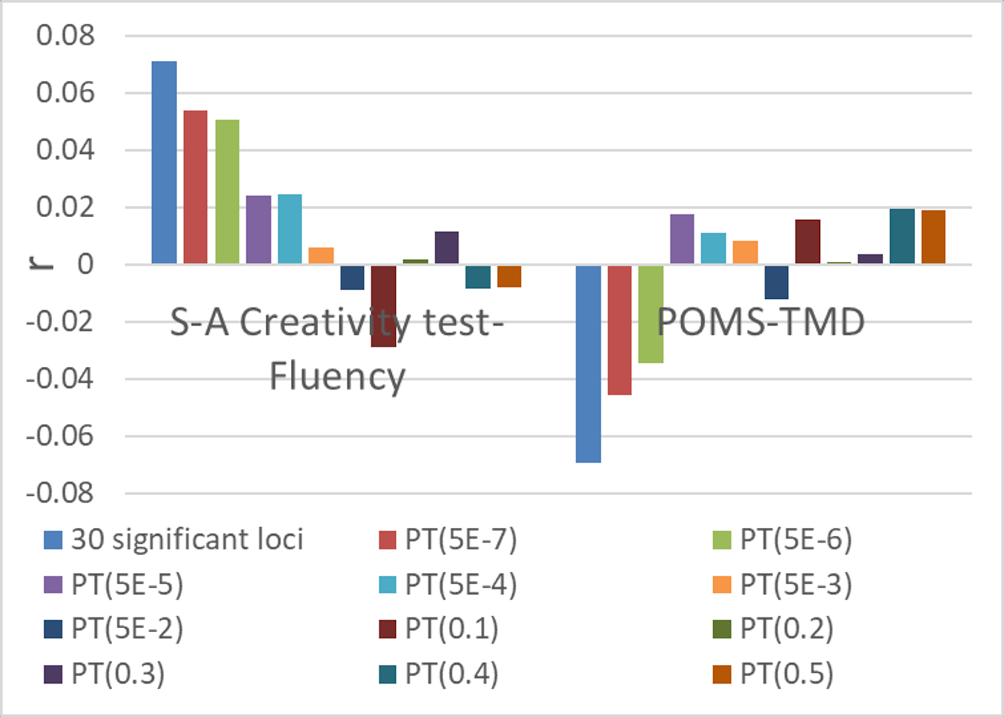


**References**

Ashburner, J., Friston, K.J. (2000) Voxel-based morphometry-the methods. Neuroimage, 11:805-821.

Browning, B.L., Browning, S.R. (2009) A unified approach to genotype imputation and haplotype-phase inference for large data sets of trios and unrelated individuals. The American Journal of Human Genetics, 84:210-223.

Chang, C.C., Chow, C.C., Tellier, L.C., Vattikuti, S., Purcell, S.M., Lee, J.J. (2015) Second-generation PLINK: rising to the challenge of larger and richer datasets. Gigascience, 4:s13742-015-0047-8.

Chavez-Eakle, R.A., del Carmen Lara, M., Cruz-Fuentes, C. (2006) Personality: A Possible Bridge Between Creativity and Psychopathology? Creativity. Res. J., 18:27-38.

Demontis, D., Walters, R.K., Martin, J., Mattheisen, M., Als, T.D., Agerbo, E., Baldursson, G., Belliveau, R., Bybjerg-Grauholm, J., Bækvad-Hansen, M. (2019) Discovery of the first genome-wide significant risk loci for attention deficit/hyperactivity disorder. Nat. Genet., 51:63-75.

Hancock, D.B., Levy, J.L., Gaddis, N.C., Bierut, L.J., Saccone, N.L., Page, G.P., Johnson, E.O. (2012) Assessment of genotype imputation performance using 1000 Genomes in African American studies. PLoS ONE, 7:e50610.

Kässens, J.C., Wienbrandt, L., Ellinghaus, D. (2021) BIGwas: Single-command quality control and association testing for multi-cohort and biobank-scale GWAS/PheWAS data. GigaScience, 10:giab047.

King, L.A., Walker, L.M., Broyles, S.J. (1996) Creativity and the five-factor model. Journal of Research in Personality, 30:189-203.

Li, H.-J., Zhang, C., Hui, L., Zhou, D.-S., Li, Y., Zhang, C.-Y., Wang, C., Wang, L., Li, W., Yang, Y. (2021) Novel Risk Loci Associated With Genetic Risk for Bipolar Disorder Among Han Chinese Individuals: A Genome-Wide Association Study and Meta-analysis. JAMA psychiatry, 78:320-330.

Miller, J.A., Scult, M.A., Conley, E.D., Chen, Q., Weinberger, D.R., Hariri, A.R. (2018) Effects of schizophrenia polygenic risk scores on brain activity and performance during working memory subprocesses in healthy young adults. Schizophr. Bull., 44:844-853.

Minds, S.F.C. (1969) Manual of S-A creativity test. Tokyo, Japan. Tokyo shinri Corporation.

Nish, Y., Niwase, K. (2003) Creative attitude of elementary students in a city and an island [in Japanese]. Bulletin of currirulum research and development, Hyogo University of Teacher Education:15-23.

Ogata, S. (1976) Evaluation of children's creative thinking ability through the figure completion task. Bulletin of Faculty of Education, Nagasaki University. Educational science, 23:45-55.

Oldfield, R.C. (1971) The assessment and analysis of handedness: the Edinburgh inventory. Neuropsychologia, 9:97-113.

Prins, B.P., Abbasi, A., Wong, A., Vaez, A., Nolte, I., Franceschini, N., Stuart, P.E., Guterriez Achury, J., Mistry, V., Bradfield, J.P. (2016) Investigating the causal relationship of C-reactive protein with 32 complex somatic and psychiatric outcomes: a large-scale cross-consortium Mendelian randomization study. PLoS Med., 13:e1001976.

Ranlund, S., Calafato, S., Thygesen, J.H., Lin, K., Cahn, W., Crespo‐Facorro, B., de Zwarte, S.M., Díez, Á., Di Forti, M., GROUP. (2018) A polygenic risk score analysis of psychosis endophenotypes across brain functional, structural, and cognitive domains. American Journal of Medical Genetics Part B: Neuropsychiatric Genetics, 177:21-34.

Reed, E., Nunez, S., Kulp, D., Qian, J., Reilly, M.P., Foulkes, A.S. (2015) A guide to genome‐wide association analysis and post‐analytic interrogation. Stat. Med., 34:3769-3792.

Shimonaka, Y., Nakazato, K. (2007) Creativity and factors affecting creative ability in adulthood and old age. Japanese Journal of Educational Psychology, 55:231-243.

Stahl, E.A., Breen, G., Forstner, A.J., McQuillin, A., Ripke, S., Trubetskoy, V., Mattheisen, M., Wang, Y., Coleman, J.R., Gaspar, H.A. (2019) Genome-wide association study identifies 30 loci associated with bipolar disorder. Nat. Genet., 51:793-803.

Takeuchi, H., Taki, Y., Nouchi, R., Hashizume, H., Sekiguchi, A., Kotozaki, Y., Nakagawa, S., Miyauchi, C.M., Sassa, Y., Kawashima, R. (2014) Effects of Multitasking-Training on Gray Matter Structure and Resting State Neural Mechanisms. Hum. Brain Mapp., 35:3646-3660.

Takeuchi, H., Taki, Y., Nouchi, R., Sekiguchi, A., Hashizume, H., Sassa, Y., Kotozaki, Y., Miyauchi, C.M., Yokoyama, R., Iizuka, K., Seishu, N., Tomomi, N., Kunitoki, K., Kawashima, R. (2015) Degree centrality and fractional amplitude of low-frequency oscillations associated with Stroop interference. Neuroimage, 119:197-209.

Takeuchi, H., Taki, Y., Nouchi, R., Yokoyama, R., Kotozaki, Y., Nakagawa, S., Sekiguchi, A., Iizuka, K., Hanawa, S., Araki, T., Makoto, M.C., Kohei, S., Yuko, S., Takayuki, N., Shigeyuki, I., Susumu, Y., Daniele, M., Ryuta, K. (2020) Originality of divergent thinking is associated with working memory–related brain activity: evidence from a large sample study. Neuroimage, 216:article 116825.

Takeuchi, H., Taki, Y., Nouchi, R., Yokoyama, R., Kotozaki, Y., Nakagawa, S., Sekiguchi, A., Iizuka, K., Yamamoto, Y., Hanawa, S., Araki, T., Miyauchi, C.M., Shinada, T., Sakaki, K., Sassa, Y., Nozawa, T., Ikeda, S., Yokota, S., Daniele, M., Kawashima, R. (2017) Creative females have larger white matter structures: evidence from a large sample study. Hum. Brain Mapp., 38:414-430.

Takeuchi, H., Taki, Y., Sassa, Y., Hashizume, H., Sekiguchi, A., Fukushima, A., Kawashima, R. (2010) Regional gray matter volume of dopaminergic system associate with creativity: Evidence from voxel-based morphometry Neuroimage, 51:578-585.

Takeuchi, H., Taki, Y., Sekiguchi, A., Nouchi, R., Kotozaki, Y., Nakagawa, S., Miyauchi, C.M., Iizuka, K., Yokoyama, R., Shinada, T., Yamamoto, Y., Hanawa, S., Araki, T., Hashizume, H., Sassa, Y., Kawashima, R. (2013) Association of hair iron levels with creativity and psychological variables related to creativity. Frontiers in Human Neuroscience, 7, Article 875:1-9.

Takeuchi, H., Tomita, H., Browne, R., Taki, Y., Kikuchi, Y., Ono, C., Yu, Z., Nouchi, R., Yokoyama, R., Kotozaki, Y. (2021) Sex-Dependent Effects of the APOE ɛ4 Allele on Behavioral Traits and White Matter Structures in Young Adults. Cereb. Cortex, 31:672-680.

Torrance, E.P. (1966) Torrance tests of creative thinking. Bensenville, IL. Scholastic Testing Service Bensenville, Ill.

Watanabe, T. (1998) A study on the individual differences of the experience of hypnagogic imagery. Shinrigaku kenkyu: The Japanese journal of psychology, 68:478-483.

Wulanqiqige. (2014) Investigation of Evaluation of Creativity for Junior High School Education. Doctor thesis: School of Knowledge Science, Japan Advanced Institute of Science and Technology.
