# Supplementary figures and images for: Polygenic risk score for bipolar disorder associates with divergent thinking and brain structures in the prefrontal cortex
Source: Hum Brain Mapp. 2021 Sep 29;42(18):6028–37. doi: 10.1002/hbm.25667 (PMC8596941; doi:10.1002/hbm.25667)

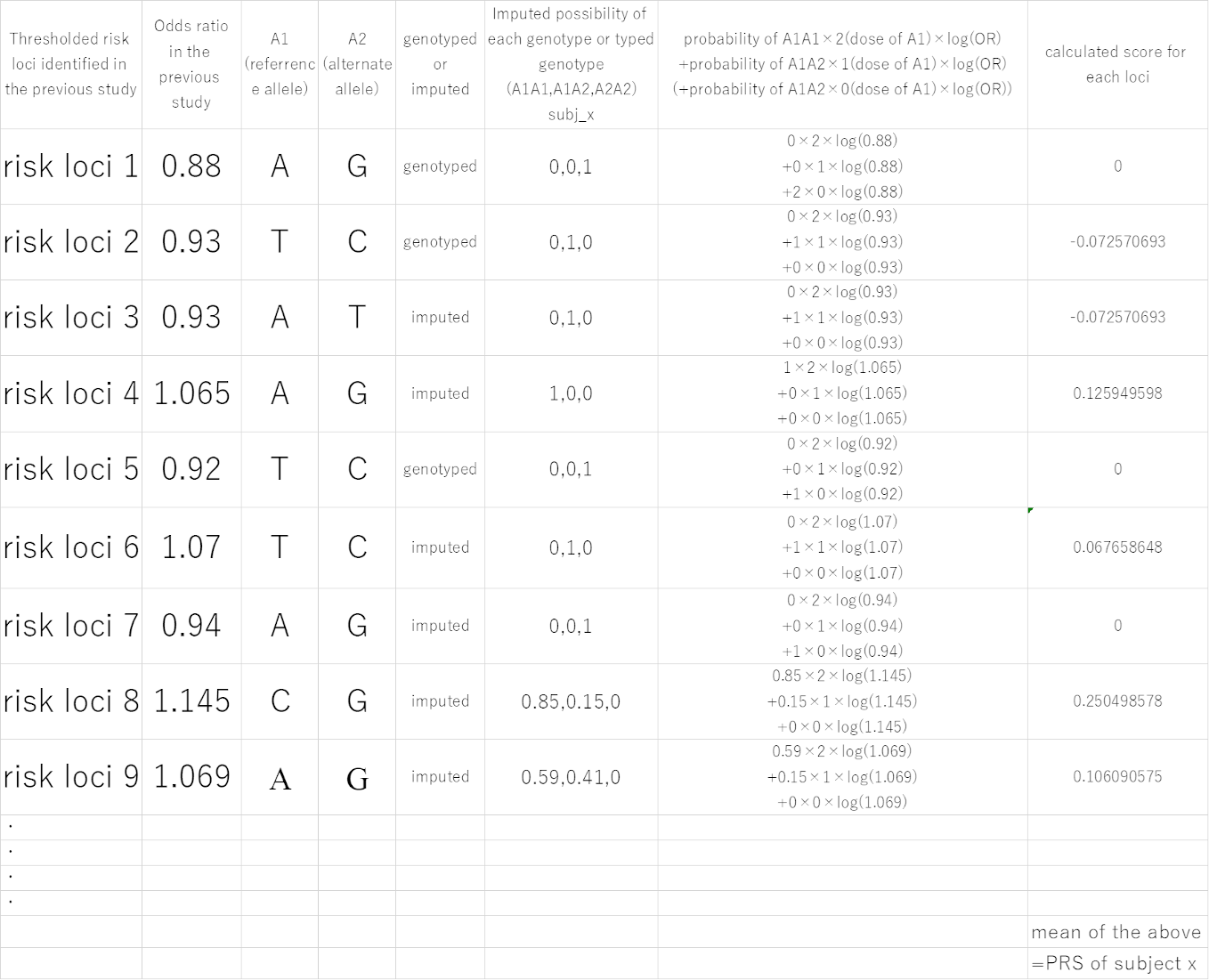

Supplement: Supplementary file 2 — Figure S1 The example schema of how BD‐PRS is calculated for each individual from genotyped and imputed information of each allele and each SNP and previously shown OR for each risk allele. The equation is as follows: w i is the natural log of odds ratio from the previous study for SNP i with w i = ln (OR i ), and P i is the probability of reference allele of each individual for that SNP. PRS=∑i=124wiPi [file HBM-42-6028-s001.tif]

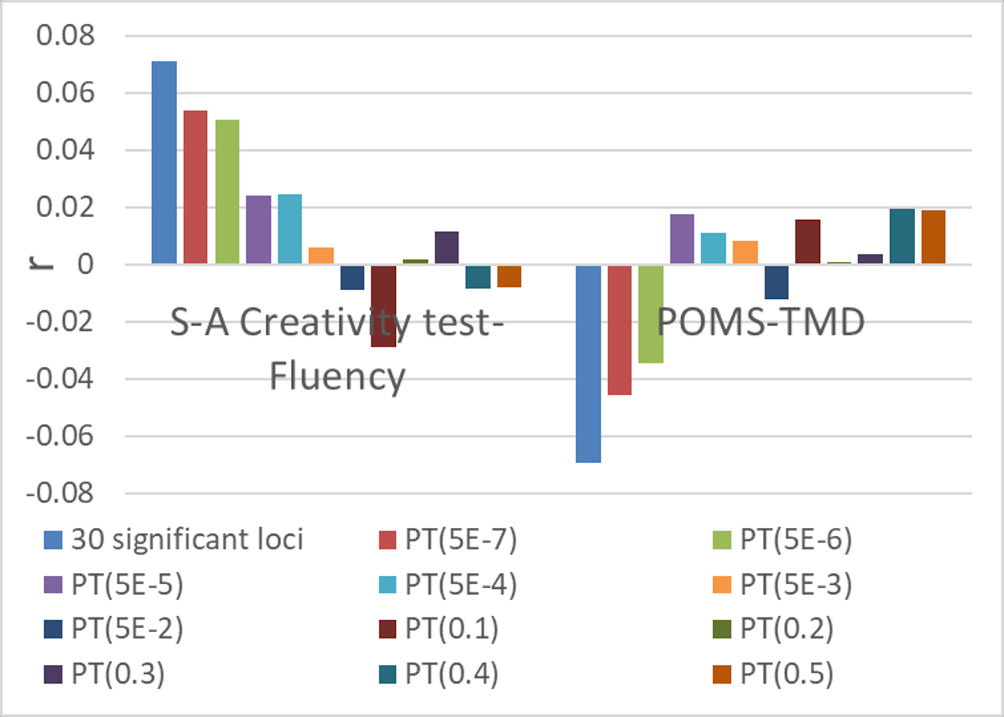

Supplement: Supplementary file 3 — Figure S2 Pearson's correlation coefficients of the correlation between two main results and the BD‐PRS of each threshold. Note among the 30 significant loci that were identified in the previous study, data from 24 loci were available in this study. The 30 significant loci in the previous study were derived from the analyses combining the discovery sample and the follow‐up sample. However, the BD‐PRS based on each p‐value's threshold (PT) was calculated from the data of the discovery sample of the previous study (available from the Psychiatric Genomics Consortium (https://www.med.unc.edu/pgc/). [file HBM-42-6028-s003.tif]
